# Transcriptional expression of ZICs as an independent indicator of survival in gliomas

**DOI:** 10.1038/s41598-021-93877-3

**Published:** 2021-09-02

**Authors:** Zhaocheng Han, Jingnan Jia, Yangting Lv, Rongyanqi Wang, Kegang Cao

**Affiliations:** Dongzhimen Hospital, Beijing University of Chinese Medicine, No.5 Haiyuncang Rd., Dongcheng District, Beijing, 100700 China

**Keywords:** Cancer, Computational biology and bioinformatics

## Abstract

The functional significance of the zinc-finger of the cerebellum (ZIC) gene family in gliomas remains to be elucidated. Clinical data from patients with gliomas, containing expression levels of ZIC genes, were extracted from CCLE, GEPIA2 and The Human Protein Atlas (HPA). Univariate survival analysis adjusted by Cox regression via OncoLnc was used to determine the prognostic significance of ZIC expression. We used cBioPortal to explore the correlation between gene mutations and overall survival (OS). ZIC expression was found to be related to immune cell infiltration in gliomas via TIMER analysis. GO term and KEGG pathway enrichment analyzes were performed with Metascape. PPI networks were constructed using STRING. The expression levels of ZIC1/3/4/5 in gliomas were significantly different from those in normal samples. High expression levels of ZIC1/5 were associated with poor OS in brain low-grade glioma (LGG) patients, while low ZIC3 expression combined was related to favorable OS in glioblastoma multiforme (GBM). ZIC alterations were associated with poor prognosis in LGG patients and related to favorable prognosis in GBM patients. We observed that the expression of ZICs was related to immune cell infiltration in glioma patients. ZICs were enriched in several pathways and biological processes involving Neuroactive ligand-receptor interaction (hsa04080). The PPI network revealed that some proteins coexpressed with ZICs played a role in the pathogenesis of gliomas. Differences in the expression levels of ZIC genes could provide a significant marker for predicting prognosis in gliomas.

## Introduction

Gliomas are the most common type of primary tumour arising from glial cells, representing 75% of malignant brain tumours^1^. The World Health Organization (WHO) divides gliomas, including low-grade gliomas (LGG) and high-grade gliomas (HGG), into 4 stages^2^. WHO stage IV glioma, also known as glioblastoma multiforme (GBM), has a remarkably poor prognosis and accounts for 2.9% of cancer-related deaths but only 1.4% of cancers^1,3^. Over the years, although great progress has been made in surgery and chemoradiation, the overall median survival of patients is approximately 12.6 months and has not improved significantly^4,5^. Existing studies have suggested that glioma progression is associated with the regulatory networks of genes^6,7^. Therefore, investigating biomarkers underlying glioma tumorigenesis might be helpful for improving therapeutic strategies.

Some studies have confirmed that aberrant expression of mRNAs is involved in the carcinogenesis process^8^. Moreover, glioblastoma cell migration and invasion were suppressed by the expression of some mRNAs^9^. Human zinc-finger of the cerebellum (ZIC) family genes are indispensable during development. A previous study showed that ZIC5, a member of the ZIC family, drives melanoma aggressiveness by activating FAK and STAT3^10^. In addition, ZIC1 could reprogramme glioma cells into neuron-like cells, which suggested that ZIC family members were associated with glioma^11^. Whether ZICs have prognostic value in glioma patients remains unclear.

To determine the role of ZICs as prognostic biomarkers in LGG and GBM, we performed a comprehensive bioinformatics analysis using multiple datasets and further explored these data to identify the role of ZICs in glioma.

## Methods

### Broad Institute Cancer Cell Line Encyclopedia

Broad Institute Cancer Cell Line Encyclopedia (CCLE) (https://portals.broadinstitue.org/ccle), an open-access database containing data on 1457 cell lines and 84,434 genes, is a resource for that uses model cancer cell lines to accelerate cancer research^12^. In this study, scatterplots for ZIC expression were obtained from CCLE.

### GEPIA2

GEPIA2 (http://www.gepia2.cancer-pku.cn/#index) is a comprehensive online platform that provides fast and customized delivery of functionalities from the genotype-tissue expression (GTEx) and TCGA (The Cancer Genome Atlas) databases^13^. In our study, we used GEPIA2 to analyze the transcriptional expression of ZIC family members among different cancer tissues and their corresponding contiguous normal control samples. The prognostic values of genes were validated using a Kaplan–Meier curve.

### The human protein atlas

The Human Protein Atlas (http://www.proteinatlas.org) includes immunohistochemistry-based expression data for almost 20 extremely common cancers, and every tumour type includes 12 individual tumour samples^14^. In our study, the gene expression of ZICs was explored using immunofluorescence data from brain-related cell lines. Next, the protein expression of ZIC1/3 in human normal tissues was determined by immunohistochemistry and compared with that in glioma tissues.

### OncoLnc

OncoLnc (https://www.oncolnc.org) contains survival data from 8647 patients from The Cancer Genome Atlas (TCGA) and includes data from 21 cancer studies. Users can download the survival data or create Kaplan–Meier plots^15^. In this study, the prognostic value of ZIC expression in LGG and GBM patients was analyzed using this tool. We downloaded the survival data of cancer patients who had been divided into low and high expression groups by median to further analyze the utility of ZICs in predicting survival.

### cBioPortal

cBioPortal (http://www.cbioportal.org) is a comprehensive web resource for the exploration and analysis of multidimensional cancer genomics data^16^. In our study, we used Glioblastoma Multiforme(TCGA, PanCancer Atlas) dataset to analyze the genomic data of ZICs, which included mRNA expression z-scores relative to diploid samples (RNASeq V2RSEM) with a z-score threshold of ± 1.8, mutations and putative copy number alterations with the GISTIC tool. Their relationship of ZIC genetic mutations with overall survival was analyzed with Kaplan–Meier plots, and the log-rank test was used to determine the significance of differences between the survival curves. A *P* value < 0.05 was considered statistically significant.

### TIMER

TIMER (http://cistrome.shinyapps.io/timer/), a user-friendly interactive web resource, identified the proportions of different immune cells based on expression data and the corresponding clinical impact of these cells for systematic evaluation^17,18^. In this study, we used the “gene module” to analyze the relationship between the infiltration of immune cells and ZIC expression levels. The “survival module” was used to further explore the association between the infiltration of immune cells, ZIC expression and clinical outcome.

### Metascape

Metascape (http://metascape.org) is a publicly available tool for gene set enrichment analysis and gene annotation^19^. In this study, we found more than 100 genes similar to ZICs via the “similar genes detection module” of GEPIA2^13^. Next, Kyoto Encyclopedia of Genes and Genomes (KEGG) pathway^20–22^ enrichment analysis and Gene Ontology enrichment analysis of ZICs were performed, providing enriched cellular component (CC), biological process (BP) and molecular function (MF) terms^23–26^. The minimum enrichment was set as 3, and a P value cutoff of 0.05 was considered statistically significant.

### STRING

STRING (http://string-db.org/) is a powerful web resource covering 5090 organisms that supports functional exploration of genome-wide experimental datasets. Users are allowed to perform gene set enrichment analysis and visualize subsets of enriched proteins as interaction networks^27^. To construct a protein–protein interaction (PPI) network of differentially expressed ZIC family members, we explored additional proteins related to ZICs with STRING. We used a minimum required interaction score cutoff of ≥ 0.7 (high confidence) to obtain the significant PPIs.

### Statistical methods

The association of ZIC mRNA expression with patient survival was analyzed by Cox regression using SPSS software version 20.0. We used this software to evaluate the effect of expression of ZIC family members on prognosis.

## Results

### Transcriptional levels of ZICs in gliomas tissues

**Figure 1:**
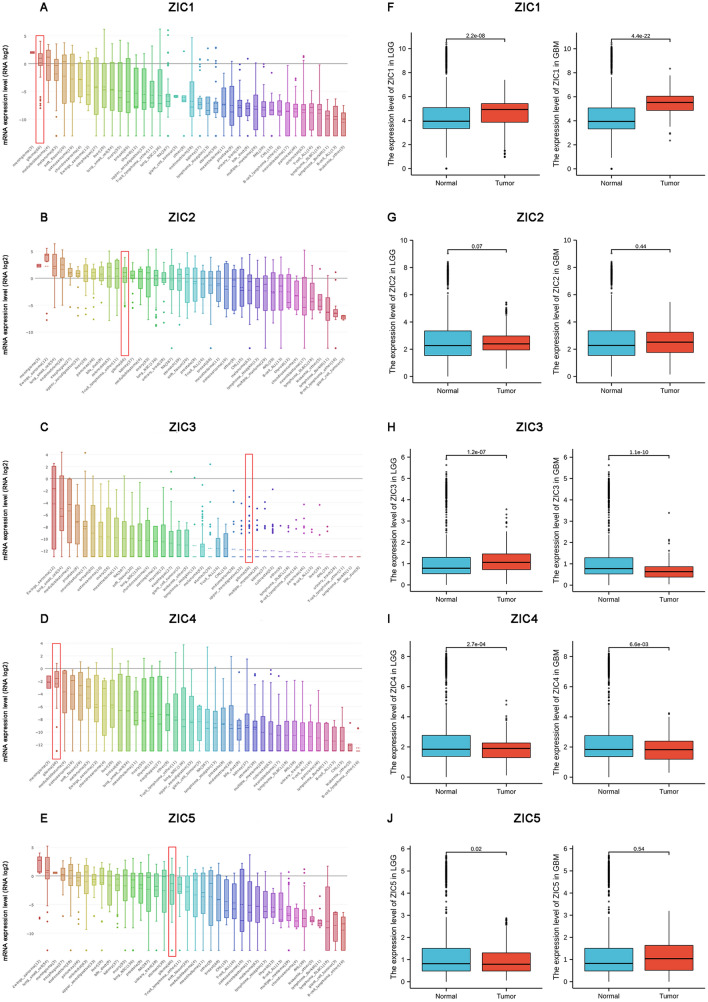
Transcriptional expression of ZICs in various types of cancer. (**A**–**E**) ZIC expression patterns in 1457 cell lines representing 40 distinct tumour types (CCLE). (**F**–**J**) mRNA expression of distinct ZIC family members in LGG tissues and adjacent normal brain tissues (GEPIA2).

As shown in Fig. 1, the mRNA expression levels of ZIC family members were measured and compared to those in corresponding normal tissues via the GEPIA2 database. Significant upregulation of ZIC1/3/4 was found in brain low-grade glioma patients, while ZIC5 was downregulated. The mRNA expression of ZIC1 were highly expressed in glioblastoma patients, and ZIC3 and ZIC4 were downregulated expression. The results regarding ZICs gene expression between the CCLE database and GEPIA2 were discrepant partly, which could have been caused by the different classifications of brain and central nervous system (CNS) cancers. Brain and CNS cancers are divided into brain low-grade gliomas and glioblastoma multiforme in GEPIA2. The expression of ZIC2 between glioma tissues and corresponding normal samples had no obvious difference (Fig. 1a–j).

**Figure 2:**
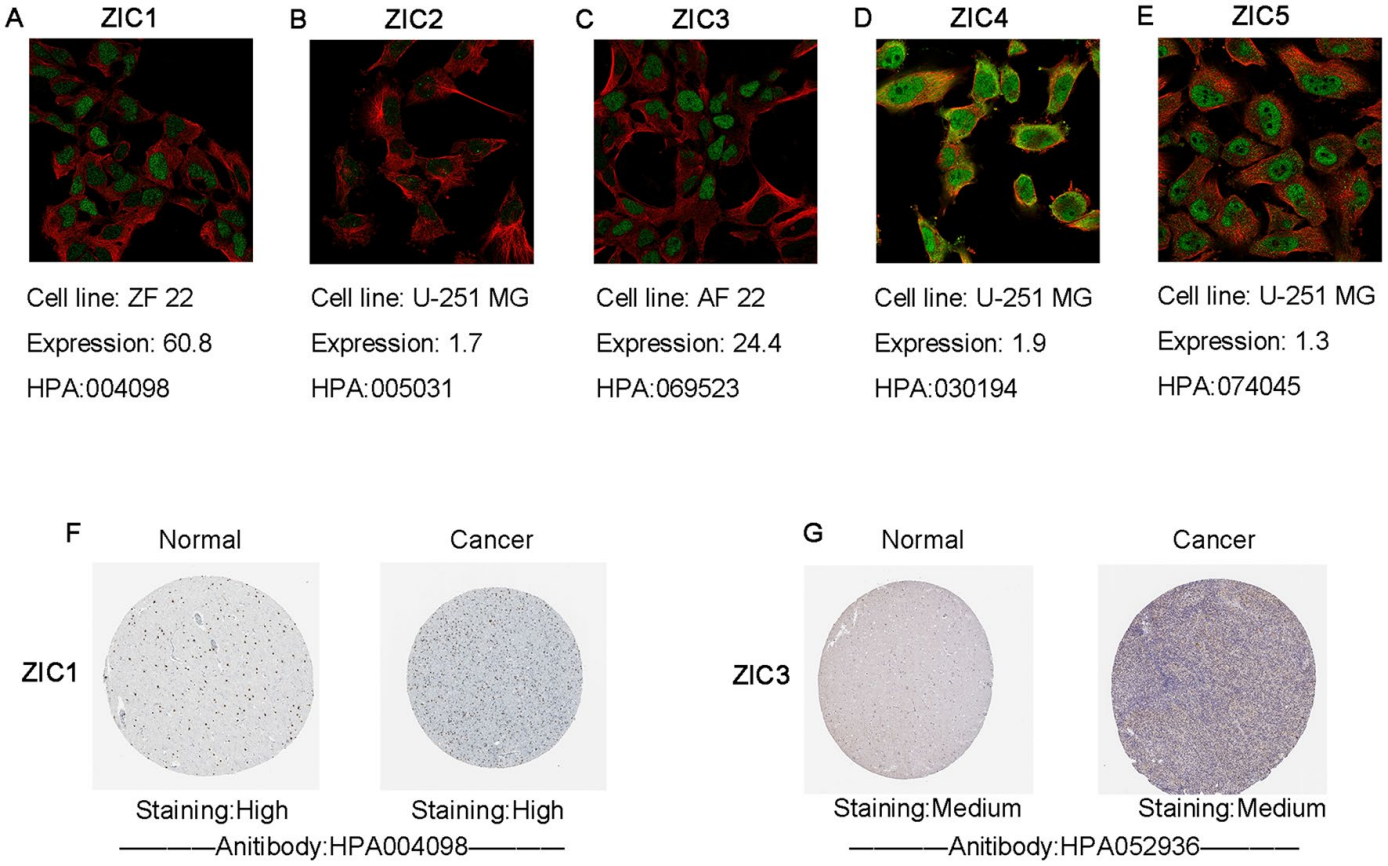
Representative immunohistochemistry images of ZICs staining in glioma tissues and normal brain tissues (HPA). Specific antibody staining is shown in green, while microtubule staining is shown in red. Brown staining indicates where an antibody has bound to its corresponding antigen in the IHC. Compared with other cell lines, expression above 1 was considered at least elevated (**A**–**E**). High ZIC1 protein expression was observed in LGG tissues (**F**), while low ZIC3 protein expression was observed in GBM tissues (**G**).

Furthermore, the gene expression of ZICs was also evaluated using immunofluorescence data from brain-related cell lines via The Human Protein Atlas (Fig. 2a–e). RNA expression above 1 was considered statistically significant. In our study, ZIC1 and ZIC3 expression were enriched in brain-related cell lines, which means in these cell lines ZIC levels are at least four times higher than any other cell type. After analyzing the mRNA expression patterns of ZICs in glioma, we further explored the protein expression of ZICs in glioma (Fig. 2f,g). According to information provided by the HPA database, ZIC1 was highly expressed in normal brain tissues, and higher expression of ZIC was observed in malignant low-grade glioma than in normal tissues. In addition, lower protein expression of ZIC3 was identified in malignant high-grade glioma tissues compared with normal samples. The protein expression data for ZIC2/4/5 in the HPA database were incomplete. Taken together, our results showed that the gene expression of ZIC1 and ZIC3 were obviously different between glioma and normal samples, and the evidence of gene protein expression was not sufficient which needed further experimental verification..

### Prognostic significance of ZIC mRNA expression in glioma patients

**Figure 3:**
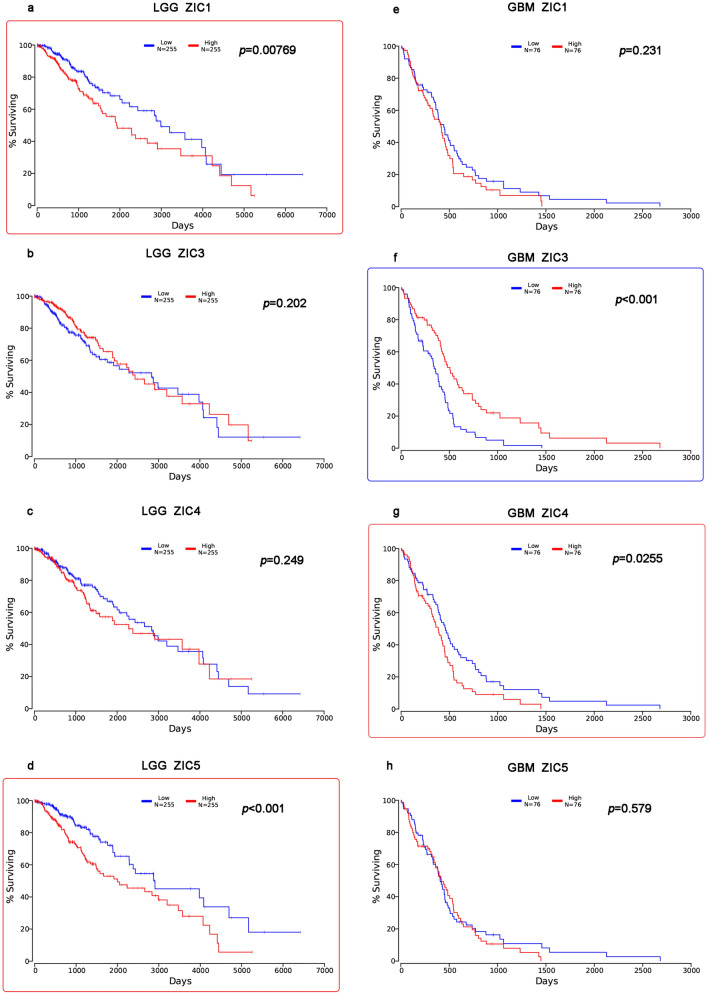
Prognostic value of ZIC family member mRNA expression in LGG and GBM patients (OncoLnc). The framed images contain parameters that were found to have prognostic relevance. High mRNA expression of ZIC1/5 individual was associated with poor OS in LGG patients (**a**,**d**). Low mRNA expression of ZIC3 was significantly associated with poor OS in GBM patients (**f**), while high expression of ZIC4 was significantly related to unfavorable OS in GBM patients (**g**). ZIC1/5 was not related to the OS in GBM patients (**e**,**h**), and ZIC3/4 was not related to the OS in LGG patients (**b**,**c**).

To explore the utility of ZICs in predicting survival, we analyzed the mRNA expression of ZICs in both LGG and GBM patients via OncoLnc. As shown in Fig. 3, the mRNA expression of ZICs was significantly associated with glioma patient prognosis. Highly expression levels of ZIC1/5 were associated with short overall survival (OS) in LGG patients. (Fig. 3a,d). Downregulated expression of ZIC3 was significantly related to unfavorable OS (Fig. 3f). Next, the patient data were downloaded to further validate these results by univariate and multiple Cox regression analysis. The outcome data are shown in Tables 1 and 2 (*p* < 0.05 was considered statistically significant). These results indicated that the mRNA expression of ZIC1/5 was a significant independent prognostic predictor for OS in LGG patients, and the mRNA expression of ZIC3 was related to GBM patient prognosis, which suggested that ZIC1/5 and ZIC3 are independent prognostic factors in LGG and GBM patients, individually.

**Table 1:** Univariate and multivariate analysis analysis of overall survival in LGG patients. (TCGA database).

	Univariate analysis			Multivariate analysis		
	HR	95% CI	p	HR	95% CI	p
Gender	0.918	0.643 ~ 1.309	0.635	1.009	0.694 ~ 1.467	0.961
Age	1.058	1.043 ~ 1.074	< 0.001***	1.060	1.043 ~ 1.076	< 0.001***
Race	1.182	0.800 ~ 0.1745	0.401	0.797	0.528 ~ 1.202	0.278
RT	0.516	0.334 ~ 0.798	0.003**	0.655	0.414 ~ 1.036	0.070
ZIC1	1.623	1.142 ~ 2.307	0.007**	1.687	1.127 ~ 2.525	0.011*
ZIC2	2.152	1.512 ~ 3.062	< 0.001***	0.907	0.542 ~ 1.518	0.711
ZIC3	0.839	0.679 ~ 1.215	0.353	0.727	0.487 ~ 1.084	0.118
ZIC4	1.270	0.880 ~ 1.831	0.201	1.001	0.662 ~ 1.513	0.995
ZIC5	1.931	1.357 ~ 2.747	< 0.001***	1.997	1.233 ~ 3.234	0.005**

*RT* Radiation therapy, *HR* hazard ration, *95% CI* 95% confidential interval.

*p < 0.05, **p < 0.01, ***p < 0.001.

Bold values indicate clinical significance.

**Table 2:** Univariate and multivariate analysis analysis of overall survival in GBM patients. (TCGA database).

	Univariate analysis				Multivariate analysis		
	HR	95%CI		p	HR	95%CI	p
Gender	1.104	0.820 ~ 1.486		0.515	0.998	0.735 ~ 1.355	0.989
Age	1.011	1.001 ~ 1.022		0.035*	1.009	0.998 ~ 1.020	0.118
Race	1.221	0.885 ~ 1.685		0.313	1.229	0.890 ~ 1.697	0.210
ZIC1	1.471	1.096 ~ 1.974		0.010*	1.425	1.013 ~ 2.005	0.042*
ZIC2	1.240	0.924 ~ 1.663		0.151	1.042	0.698 ~ 1.557	0.840
ZIC3	0.685	0.497 ~ 0.942		0.020*	0.687	0.493 ~ 0.956	0.026*
ZIC4	1.241	0.923 ~ 1.667		0.152	0.991	0.705 ~ 1.394	0.959
ZIC5	1.304	0.974 ~ 1.747		0.075	1.229	0.829 ~ 1.822	0.305

*HR* hazard ration, *95% CI* 95% confidential interval.

*p < 0.05, **p < 0.01, ***p < 0.001.

Bold values indicate clinical significance.

### Genetic mutation in ZICs and its relationship with the OS of glioma patients

**Figure 4:**
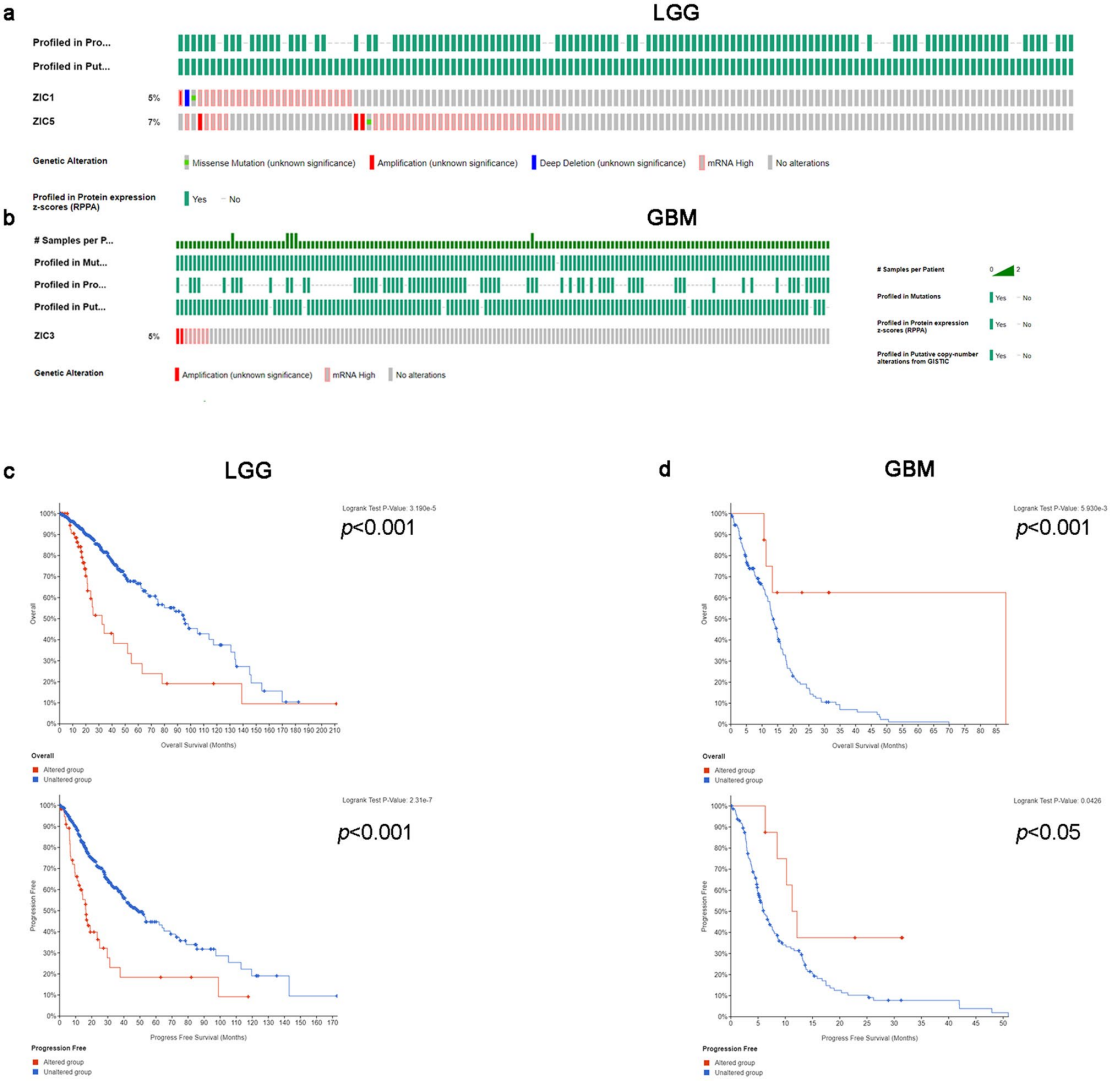
Genetic mutations in ZICs and their relationship with the OS of gliomas patients (cBioPortal). The mutation rates of ZIC1and ZIC5 were 0.39% in LGG (**a**), while they were no mutations in GBM (**b**). Genetic alterations in ZICs were associated with poor OS and PFS in LGG patients (**c**) and favorable OS and PFS in GBM patients (**d**).

Next, we analyzed genetic alterations in ZICs and their correlation with the overall survival of LGG and GBM patients. In the 514 sequenced LGG patient samples, gene alterations were observed in 59 samples, and the mutation rates for ZIC1/5 were 0.39%, (Fig. 4a). Meanwhile, in 155 GBM patients, gene alterations were found in 8 patients with no mutated cases, which implied that mutation or DNA copy-number alteration of ZICs occurs at a low rate in LGG and GBM (Fig. 4b). Next, the results from the log-rank test and Kaplan–Meier plots showed that genetic alteration in ZICs was associated with poor OS and PFS (Progression-free survival) in LGG patients (Fig. 4c) and favorable OS in GBM patients (Fig. 4d). These results suggested that genetic alteration of ZICs could significantly influence LGG and GBM patient prognosis (all *p* < 0.05) (Supplementary Figure 1).

### Relationship between immune cell infiltration and ZIC expression in patients with gliomas

**Figure 5:**
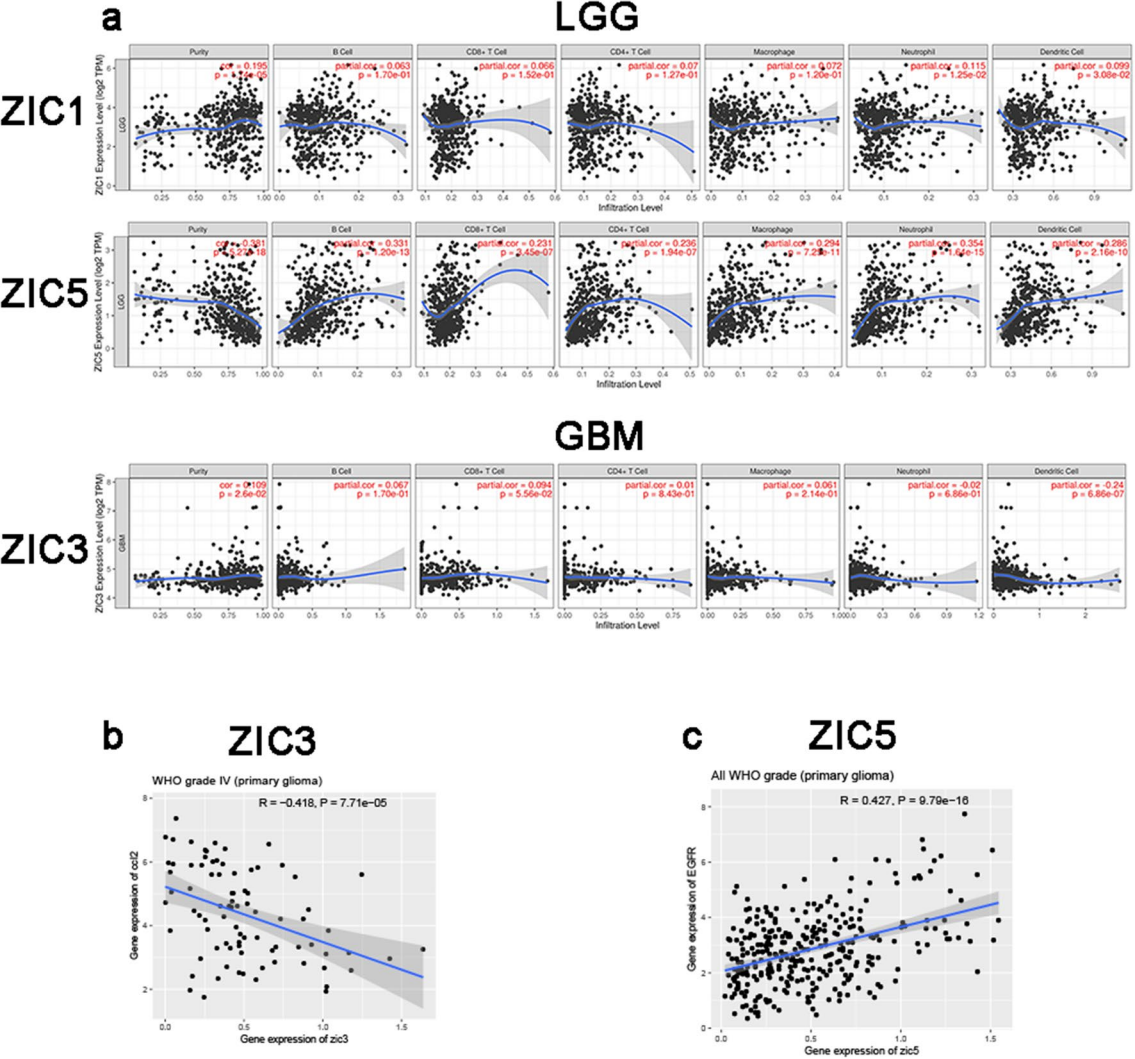
The association between differentially expressed ZICs and immune cell infiltration in gliomas samples (**A**,**B**) (TIMER).

ZICs are involved in immune cell infiltration and inflammatory responses, and hence, the clinical outcome of glioma patients related to these factors should be considered. We explored the association between immune cell infiltration and ZIC expression via the TIMER database. When summarizing the outcome data, we also took the tumour purity into account^28,29^. In LGG patients, ZIC5 expression was positively associated with infiltration of B cells, CD8+ T cells, CD4+ T cells, macrophages, neutrophils, and dendritic cells (Fig. 5a, Tables 3 and 4) *p* < 0.05 was considered statistically significant.

**Table 3:** Cox proportional hazards model factors related to survival, immune infiltrates and genes in LGG patients.

	Coefficient	HR	95% CI	*p*-value
Purity	0.225	1.253	0.452–3.476	0.665
B cells	2.454	11.639	0.012–11,143.197	0.483
CD8 + T cells	6.406	605.384	0.583–628,665.798	0.071
CD4 + T cells	4.167	64.532	0.016–254,819.792	0.324
Macrophages	4.659	105.554	1.658–6613.343	0.027*
Neutrophils	− 9.562	< 0.001	0.000–0.333	0.027*
Dendritic cells	0.552	1.737	0.020–147.756	0.808
ZIC1	0.363	1.438	1.093–1.891	0.009**
ZIC2	0.973	2.647	1.742–4.020	< 0.001***
ZIC3	− 0.647	0.524	0.327–0.839	0.007**
ZIC4	− 0.148	0.862	0.546–1.361	0.525
ZIC5	− 0.662	0.516	0.316–0.841	0.008**

*HR* hazard ration, *95% CI* 95% confidential interval.

*p < 0.05, **p < 0.01, ***p < 0.001.

Bold values indicate clinical significance.

**Table 4:** Cox proportional hazards model factors related to survival, immune infiltrates and genes in GBM patients.

	Coefficient	HR	95% CI	*p*-value
Purity	− 0.562	0.570	0.117–2.774	0.486
B cells	− 1.227	0.293	0.039–2.201	0.233
CD8 + T cells	0.153	1.165	0.326–4.167	0.815
CD4 + T cells	3.215	24.915	1.856–334.364	0.015*
Macrophages	0.231	1.26	0.091–17.386	0.863
Neutrophils	− 1.078	0.34	0.017–6.992	0.485
Dendritic cells	0.340	1.406	0.471–4.195	0.542
ZIC1	0.209	1.233	0.878–1.732	0.227
ZIC2	< 0.001	1.000	0.544–1.841	0.999
ZIC3	− 1.223	0.294	0.152–0.571	< 0.001***
ZIC4	0.119	1.126	0.730–1.737	0.591
ZIC5	− 0.281	0.775	0.347–1.640	0.477

*HR* hazard ration, *95% CI* 95% confidential interval.

*p < 0.05, **p < 0.01, ***p < 0.001.

Bold values indicate clinical significance.

In TCGA database, we determined the correlation between ZIC3 and CCL2, (Fig. 5b) which participate in recruitment of macrophages. CCL2 mRNA levels could be enhanced in low-expressed ZIC3 patients. The EGFR, which constitutively activates and enhances tumorigenicity through RAS-SHC-GRB2 pathway, was correlative with ZIC5 as shown in Fig. 5c.

### Functional enrichment analysis of ZICs

**Figure 6:**
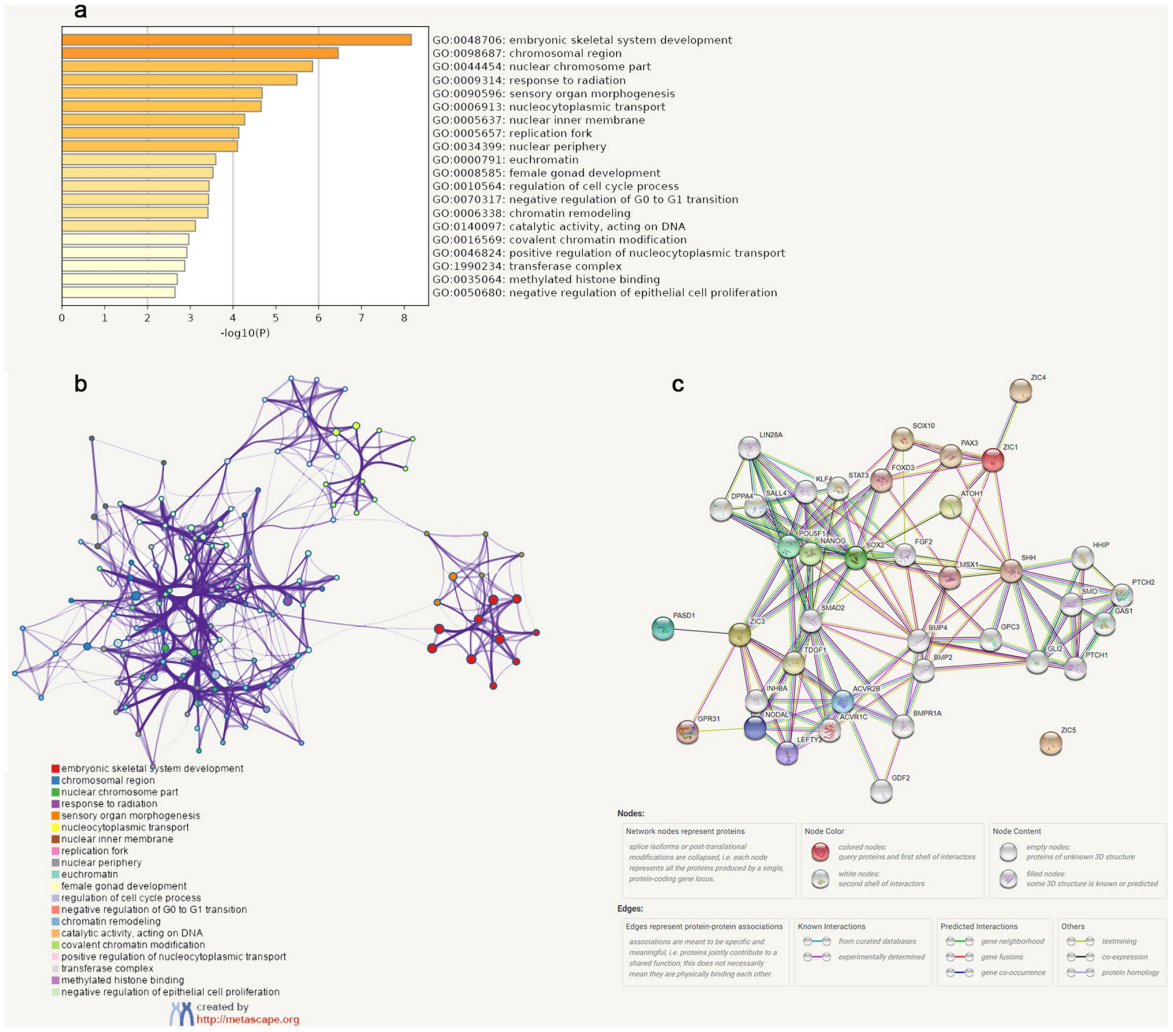
Enrichment analysis of ZIC1 and similar genes in glioma via Metascape. Heatmap of enriched terms across ZIC1, coloured by p-values (**a**). Interactive networks of enriched terms (**b**). B Distinct colours represent different pathway enriched by ZIC-correlated genes. The PPI networks of ZIC1 and ZIC3 (**c**).

After exploring the immune cell infiltration of ZICs in glioma patients, functional enrichment analysis were construct using the genes that were coexpressed with ZIC1/5 and ZIC3 in LGG and GBM, individually. As shown in Fig. 6a, biological processes such as embryonic skeletal system development (GO: 0048706), response to radiation (GO: 0009314), and sensory organ morphogenesis (GO: 0090596) were significantly regulated by ZIC1 alterations in glioma. Cellular component terms, including chromosomal region (GO: 0098687) and nuclear chromosome part (GO: 0044454), were associated with ZIC alterations. In KEGG analysis, Neuroactive ligand-receptor interaction (hsa04080) was related to the functions of ZIC5 alternations in glioma (Fig. 6a,b) (Supplementary Figure 2).

### Construction of the PPI network

PPIs of ZIC family members were determined based on their correlation coefficients by STRING. As shown in Fig. 6c, PAX3, which has been implicated in glioma tumorigenesis is co-expressed with ZIC1. Transcriptional regulation was mutually affected by ZIC1 and SHH. ZIC3 acts as a transcriptional activator or is activated in PPIs involved in neurogenesis. We used a minimum required interaction score cutoff of ≥ 0.7 (high confidence) to obtain the significant PPIs, and the topological parameters of the PPI networks are shown in Table 5.

**Table 5:** Topological parameters for the PPI network.

Topological parameters	Calculated values
Number of nodes	39
Number of edges	148
Average node degree	7.28
Average local clustering coefficient	0.666
PPI enrichment *p*-value	< 1.0e−16

## Discussion

Gliomas are the most prevalent malignant brain tumours. There has been significant progress in understanding the biology and molecular pathogenesis of glioblastoma, but translation of these findings into improved outcomes needs more time. ZICs participate in the progression of various cancers, including bladder cancer, prostate cancer, gastric cancer and melanoma^10,30–32^. Although some ZICs have been verified to play important roles in glioma, the distinct roles of ZIC family members in glioma remain to be elucidated^33^.

In the present study, we used multiple bioinformatics analysis tools to demonstrate that ZIC family members are related to the prognosis of glioma patients. We observed a significant difference in the expression of ZICs between glioma tissues and normal samples. Upregulated expression of ZIC1/5 was confirmed to be a significant predictor of poor OS in LGG patients. Low expression of ZIC3 was a significant prognostic factor in GBM patients. Furthermore, mutation of ZICs was identified in glioma patients, and genetic alteration in ZICs was associated with short OS in LGG patients and favorable OS in GBM patients. These differences between LGG and GBM related to ZIC3 expression confirm that the expression of ZIC3 is negatively correlated with the prognosis of GBM.

Some studies have reported that dendritic cells, which have the ability to induce antitumour immunity, possess immediate tumoricidal activity^34^. Macrophages are also the most common cell type related to tumour-infiltrating immune cells^35,36^. In this study, we explored the association between the expression of ZIC family members and the infiltration of immune cells, including B cells, CD4+ T cells, CD8+ T cells, neutrophils, macrophages and dendritic cells. The results revealed that ZIC5 had weak, but statistically significant correlations with the infiltration of immune cells, which indicated that ZIC5 reflect immune status and deserve further research.

GO enrichment and KEGG pathway analyzes of ZIC1/3/5 and their 100 similar genes were performed, and our results showed that the embryonic skeletal system development, response to radiation, sensory organ morphogenesis, chromosomal region and nuclear chromosome part were significantly associated with ZIC alterations. Pathways such as Neuroactive ligand-receptor interaction were found to be regulated by ZIC3 in glioma patients.

Finally, a PPI network was generated to analyze the correlation between the expression of ZICs and their related proteins. NANOG, SOX2 and POU5F1, three stemness-associated genes that are part of the FA-BSA-NP delivery system, could facilitate autophagy inhibition and chemotherapy efficacy in glioma therapy^37^. PASD1, a coexpression gene of ZIC3, promotes glioma cell proliferation by inhibiting apoptosis in vitro^12^. ZIC proteins are critical in cerebellar development, and the ZIC-related autoimmune response impacts the pathogenesis of neurologic disorders^38^. These results suggested that ZIC proteins may play an indispensable role in the progression of gliomas.

Unfortunately, there were some limitations in the present study. Although the mRNA expression levels of different ZIC family members were associated with the OS of glioma patients, all the data were obtained from online databases in our analysis, and thus, further studies consisting of more samples are required to confirm our findings; in addition, the clinical utility of ZIC members in glioma treatment needs to be explored. Moreover, our transcriptional level analysis of immune cells reflected some aspects of the immune status, but the global changes should be considered in future studies. Further studies are needed to investigate the relationships between specific ZICs and glioma (Supplementary Information).

## Conclusion

In summary, a bioinformatics analysis was performed to confirm the prognostic value of ZIC family members and explore their mutations and relationship with immune cell infiltration. Via the CCLE, GEPIA2, HPA, cBioPortal and TIMER databases, ZIC1, and ZIC5 were identified as prognostic factors in LGG patients, and ZIC3 was identified as prognostic factors in GBM patients, and these results were replicated by univariate and multivariate Cox regression. PPI networks were constructed to analyze the correlations and shared functions between the ZICs and related proteins. Our results identified ZIC1/3/5 as novel biomarkers and potential therapeutic targets for glioma.

## Supplementary Information

Supplementary Information.

## Data Availability

The datasets generated and analyzed during this study are available.

## Abbreviations

GBM: Glioblastoma multiforme
LGG: Brain low-grade glioma
CNS: Central nervous system
OS: Overall survival
PPI: Protein–protein network
GO: Gene ontology
KEGG: Kyoto encyclopedia of genes and genomes
ZIC: Zinc-finger of the cerebellum
