# Supplementary material for: Transcriptional expression of ZICs as an independent indicator of survival in gliomas
Source: Sci Rep. 2021 Sep 2;11:17532. doi: 10.1038/s41598-021-93877-3 (PMC8413274; doi:10.1038/s41598-021-93877-3)

SF1. Genetic alterations in ZICs were associated with poor DFS in LGG patients.


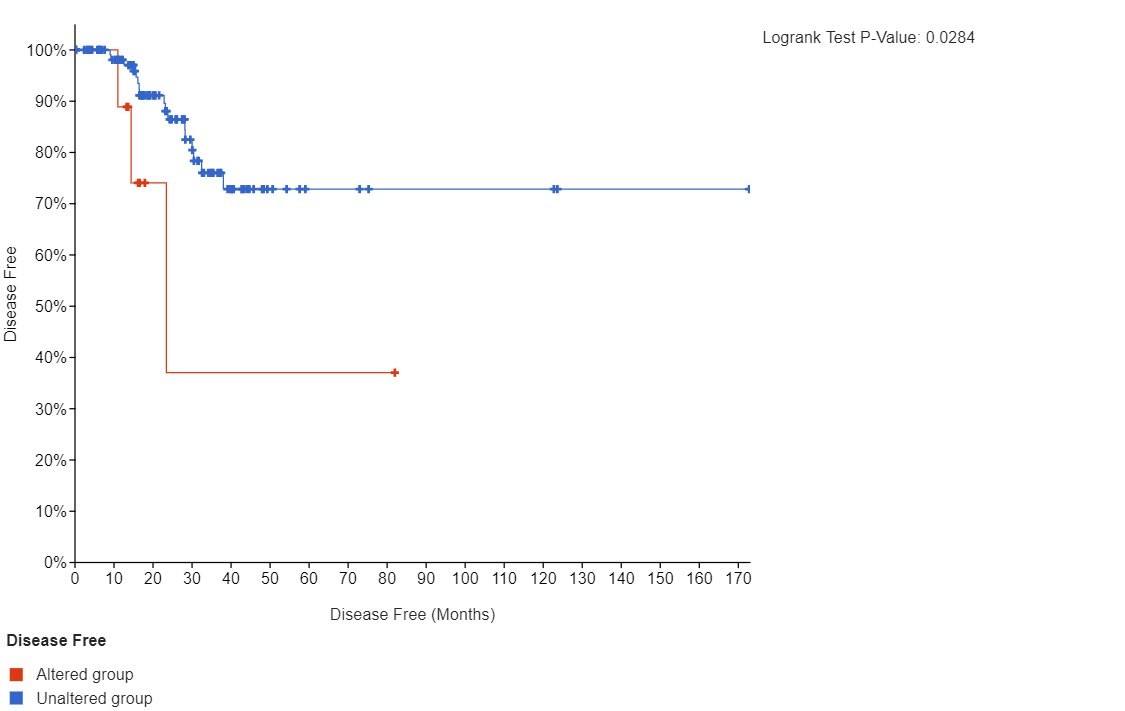


SF2. Enrichment analysis of ZIC3/5 and similar genes in glioma via Metascape. Heatmap of enriched terms across ZIC3/5, coloured by p-values(Top). Interactive networks of enriched terms(Bottom).


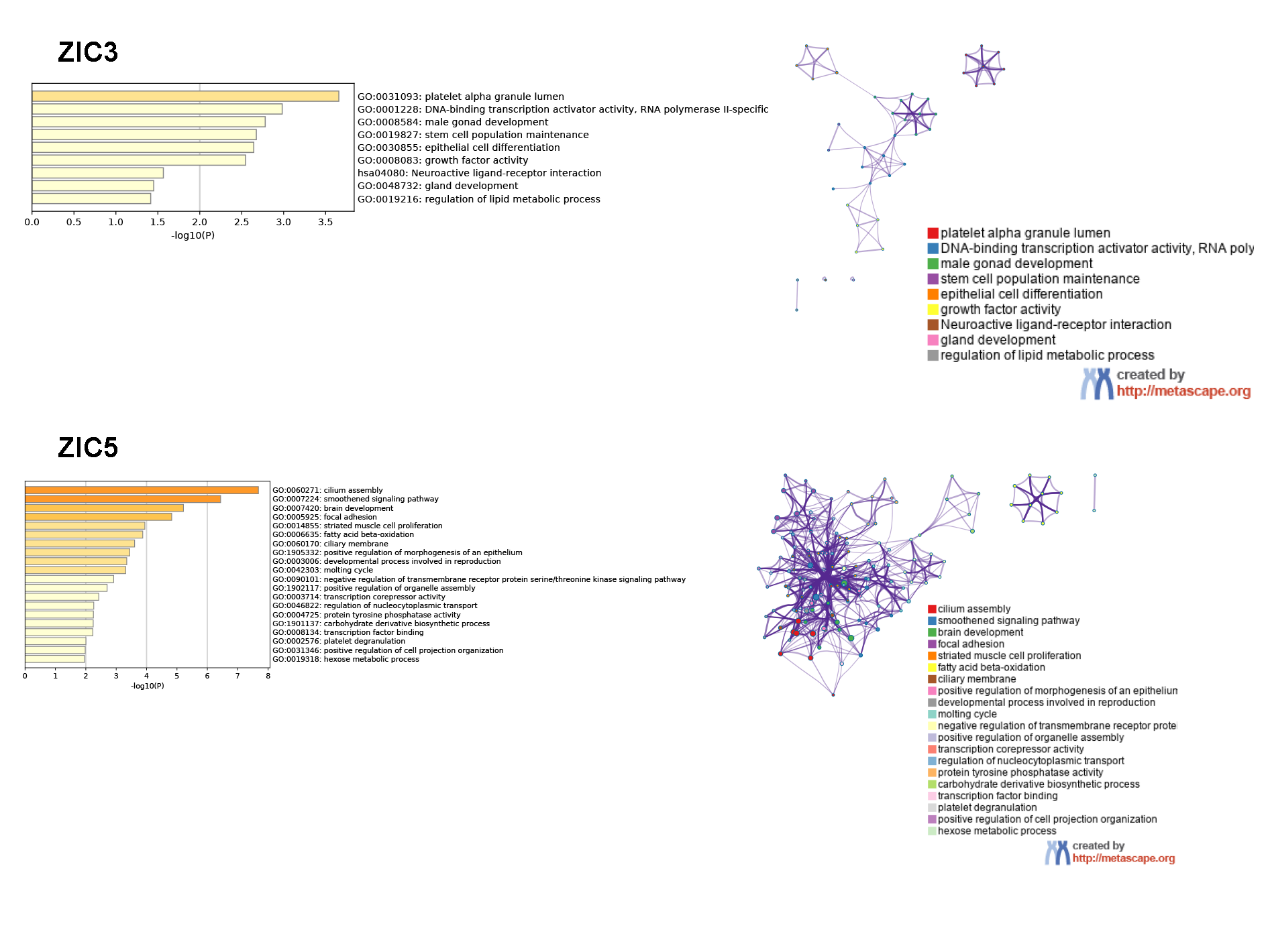

Supplement: Supplementary file 1 — Supplementary Information. [file 41598_2021_93877_MOESM1_ESM.docx]
